# Occupational ultraviolet exposure and risk of non-Hodgkin’s lymphomas: a meta-analysis

**DOI:** 10.18632/oncotarget.18140

**Published:** 2017-05-24

**Authors:** Demin Lu, Fei Xu, Kaiming Hu, Li Yin, Huijie Duan, Jiaojiao Zhang, SuZhan Zhang

**Affiliations:** ^1^ Cancer Institute (Key Laboratory of Cancer Prevention and Intervention, China National Ministry of Education, Key Laboratory of Molecular Biology in Medical Sciences), The Second Affiliated Hospital, Zhejiang University School of Medicine, Hangzhou, Zhejiang, China; ^2^ Department of Medical Oncology, The Second Affiliated Hospital, Zhejiang University School of Medicine, Hangzhou, Zhejiang, China; ^3^ Research Center for Air Pollution and Health, Zhejiang University School of Medicine, Hangzhou, Zhejiang, China

**Keywords:** occupational ultraviolet exposure, non-Hodgkin lymphoma, meta-analysis

## Abstract

Non-Hodgkin lymphoma is a heterogeneous group of lympho-proliferative disorders. We performed a meta-analysis to summarize the available evidence from case-control studies and cohort study on the inconsistent association between occupational sun exposure and the risk of non-Hodgkin lymphoma. We searched PubMed, ISI web of science, the Cochrane Library, EMBASE and reference lists for relevant articles. Study specific odds ratios or relative risk and 95% confidence intervals were pooled by using fixed-effects or random-effects models. Ten case-control studies and one cohort study were included in the meta-analysis. Overall, the pooled odds ratios for occupational ultraviolet exposure and non-Hodgkin lymphoma risk was 1.15(95% confidence intervals: 0.99, 1.32; *I*^2^ = 44.4%). Occupational sun exposure was positively associated with the risk of NHL 1.14 (95% confidence intervals: 1.05, 1.23; *I*^2^=25.4% p for heterogeneity =0.202) in Caucasian population. Common subtypes of non-Hodgkin lymphoma and ultraviolet exposure had the negative results. The pooled odds ratios was 1.16, (95%confidence intervals: 0.90, 1.50) for T-cell non-Hodgkin lymphoma; 0.79, (95%confidence intervals: 0.61, 1.02) for B-cell non-Hodgkin lymphoma; 1.13, (95%confidence intervals: 0.96, 1.34) for chronic lymphocytic leukemia; 1.25, (95%confidence intervals: 0.95, 1.64) for males; 1.49, (95%confidence intervals: 0.99, 2.25) for females. Data suggested that occupational ultraviolet exposure was a risk factor for non-Hodgkin lymphoma in Caucasian population. While, there had no relationship between occupational ultraviolet exposure and risk of non-Hodgkin lymphoma in general population as well as non-Hodgkin lymphoma common subtypes. Besides, gender specific occupational sun exposure also indicated no association on risk of non-Hodgkin lymphoma.

## INTRODUCTION

Non-Hodgkin lymphoma (NHL) is a heterogeneous group of lympho-proliferative disorders [1]. As statistic data showed, NHL is the sixth most common cancer in the United States and in the United Kingdom [2, 3], and is estimated to be the tenth most common cancer worldwide [3].

The incidence of NHL increased dramatically between 1970 and 1995 .This remarkable rise suggests a major role for environmental factors in the etiology of NHL, thus the hypothesis that solar ultraviolet(UV) radiation may explain this trend emerged in 1992 [4].

As the increasing incidence of NHL mounting studies associated with the relationship between sun exposure and risk of NHL were carried out in westernized countries. But the results have been inconsistent. We therefore undertook a meta-analysis of case-control and cohort studies to quantitatively assess the relationship between occupational sun exposure and risk of NHL and common NHL subtypes.

## RESULTS

### Literature search

**Figure 1:**
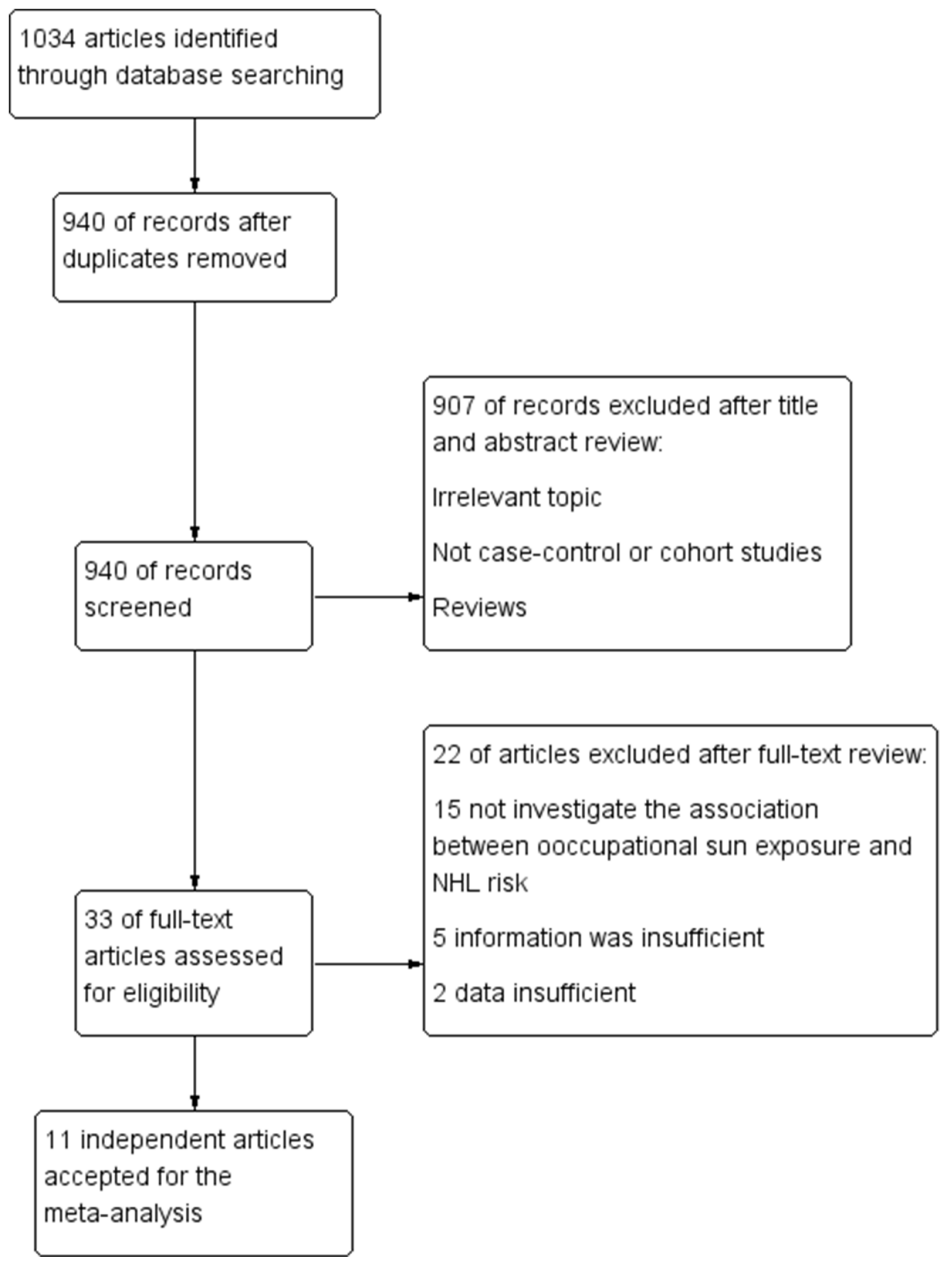
Flowchart of selection of studies for inclusion in the meta-analysis

Our systematic literature search details were shown in (Figure 1) based on search strategy and inclusion criteria. We identified 1034 abstracts from PubMed, ISI Web of Science, the Cochrane Library and EMBASE. After removing duplication 940 abstracts were remained. Of these 940 abstracts, 907 were excluded after screening titles and abstracts. The remaining 33 of them were extracted for further assessment. Twenty-two articles were excluded after full-test review. Fifteen articles were excluded because they did not investigate the association between sun exposure and NHL risk [5-19], five was insufficient information [20-24], and two was the duplicate report on the same study population [25, 26]. Thus, ten case-control studies [27-35, 37] and one cohort study [36] were eligible for our meta-analysis.

### Study characteristics

The characteristics of the 11 eligible studies were summarized in (Table 1). All studies were published between 1997 and 2012. Overall, the studies included 8829 NHL patients out of 336,557 participants. Of the 11 independent studies, ten were case-control studies [27-35, 37] and one was cohort study [36]. Two studies (one case-control and one cohort study) were conducted in Sweden [27, 36], two in Australia (two case-control studies) [28, 31], two multi-countries study in Europe (two case-control studies) [34, 37] one each in Italy [30], Sweden and Denmark [29], Germany [32], USA [33], and one in Singapore with Asian population [35].

**Table 1:** Study features of 11 included studies

Reference	Study type	Location	Study period	Age range	Case (participation)/control (participation)	Type of source	Assessment of measures
Nordstrom 1997 [27]	Case-control	Sweden	1987-1992	---	121(--)/484(--)	National population registry	Mailed questionnaire; telephone interview
Hughes 2004 [28]	Case-control	Australia	2000-2001	20-74	704(85%)/694(61%)	Electoral rolls	Self-administered questionnaire; telephone interview
Smedby 2005 [29]	Case-control	Denmark and Sweden	1999-2002	18-74	3055(81%)/3187(71%)	Population	Telephone interview
Morales 2006 [37]	Case-control	Europe	1995-1997	35-69	76(91.6%)/2094(--)	Population registries or electoral rolls	Face-to-face interview
Tavani 2006 [30]	Case-control	Northern Italy	1985-1997	18-79	446(>97%)/1295(>97%)	Patients hospitalized with other conditions	Personal interview
Karipidis 2007 [31]	Case-control	Australia	2000-2001	20-74	694(85%)/694(61%)	Population, electoral rolls	Self-administered questionnaire; telephone interview
Weihkopf 2007 [32]	Case-control	Germany		18-80	589(87.4%)/589(51.4%)	Population registers	Face-to-face interview
Zhang 2007 [33]	Case-control	USA	1996-2000	21-84	601(72%)/706(--)	Population	In person interview
Boffetta 2008 [34]	Case-control	Europe	1998-2003	>17	1518(88%)/2124(81% in hospital controls, 52% in population controls)	Population registers; Patients hospitalized with other conditions	In person interview
Wong 2012 [35]	Case-control	Singapore	2004-2008	>18	541(--)/830(--)	Patients hospitalized with other conditions	Face-to-face interview

Reference	Study type	Location	Follow-up years	Case/cohort	Cohort source	Assessment of measures
Hakansson 2001 [36]	Cohort	Sweden	1971-1993	484/323860	Nationwide occupational health service program of the Swedish construction industry	Used data files from nationwide occupational health service organization

One study included only women [33] and one study included only men [36], while the rest of the studies did not specify with gender. One study reported the Asian population [35], while the rest of studies focused on Caucasian population. Nine studies reported results for all types of NHL patients, while two studies only included specific types of NHL. The Sweden study included only hairy cell leukemia cases [27]. One European study included only mycosis fungoides cases [37]. The control source of eight studies were population based [27-29, 31-33, 36, 37], two studies were hospital based [30, 35], while one study included both population control and hospital based control source [34]. Six studies’ data collection method was in-face interview [30, 32-35, 37], while four studies’ data collection method was though self-administered questionnaire and followed by a telephone interview [27-29, 31]. One study used population occupational health service program data [36].

The age of participants were all aged 17 and above. The exposure odds ratios (ORs)/relative risk (RRs) of NHL, the adjustments made for confounding and occupational history assessment were shown in Table 2.

**Table 2:** Adjustments and occupational history assessment reported by single study in this meta-analysis

Reference	Study type	OR (95% CI)	Adjustments	Occupational history assessment
Nordstrom 1997 [27]	Case-control	HCL 2.2 (1.2-3.8)	Age, sex and country	All occupations lasting longer than 1 year were classified according to the Nordic Working Classification System (NYK) 1989.
Hughes 2004 [28]	Case-control	NHL 1.21 (0.87-1.69) Men 1.20 (0.81-1.78) Women 1.27 (0.73-2.23)	Age, sex, state of residence, ethnicity, skin color and ability to tan	Hours of occupational sun exposure. For each job recorded in the calendar, data were collected about the number of days worked per week, hours worked per day and hours worked outdoors per day. Occupational hours of exposure were totalled for 50 weeks a year, assuming 2 weeks for vacations and sick leave
Smedby 2005 [29]	Case-control	NHL 1.1 (1.0-1.2) CLL 1.1 (0.9-1.3) DLBCL 1.2 (1.0-1.4) FL 0.7 (0.5-0.9) T-NHL 1.2 (0.9-1.7)	Age, sex, country and skin reaction to sun	A standardized and computer-aided questionnaire, outdoor occupation lasting 1 year or more (ever/never)
Morales 2006 [37]	Case-control	MF 2.3 (0.9-6.2)	Age, sex, region, exposure to aromatic halogenated hydrocarbons	A structured questionnaire. The type of occupation and industry was asked for each job, including the year the job started and ended. Recorded work tasks, job title, and working hours per week for each occupational period. The specific nature of the work also was addressed, such as machines or products used, duration of their use (hours per week), and dates of job tenure.
Tavani 2006 [30]	Case-control	NHL 0.96 (0.66-1.40)	Age, sex, area of residence, education and smoking	A structured questionnaire, study participants were asked whether they had been exposed to UV radiation at work and for how long
Karipidis 2007 [31]	Case-control	NHL 1.32 (0.96-1.81)	Age, sex, region of residence, ethnic origin	The questionnaire included a lifetime calendar that was used to obtain a detailed occupational history from each subject, including information about job title, employer, industry, start and finish years, number of hours worked per day and number of days worked per week
Weihkopf 2007 [32]	Case-control	T-NHL 0.9 (0.3-3.5) B-NHL 0.9 (0.6-1.4)	Age, sex, region, smoking (packyears) and alcohol consumption	A complete occupational history, including every occupational period that lasted at least 1 year. For every job held, information was obtained about the start and the end of the job phase, about job title, industry and specific job tasks. Study subjects having held potentially hazardous jobs were additionally asked to reply to job task-specific supplementary questions.
Zhang 2007 [33]	Case-control	NHL WOMEN 1.8 (1.0-3.4)	Age, race, family history of NHL, highest educational level, eye color and skin type	A standardized, structured questionnaire. For the history of occupational exposure to ultraviolet radiation, subjects were asked to provide all job titles and main duties that they had for 1 year or longer before diagnosis (for cases) or interview (for controls). Each job title was designated as indoor (purely indoor or mixed type) or outdoor. If an individual had both indoor and outdoor jobs, she was assigned to the outdoor job category.
Boffetta 2008 [34]	Case-control	NHL 1.08 (0.74-1.56) CLL 1.36 (0.86-2.14) DLBCL 0.69 (0.42-1.15) FL 0.57 (0.31-1.06) T-NHL 1.14 (0.59-2.21)	Age, sex, study area, education, skin reaction to sun and questionnaire type	Information on occupation was collected at interview for each job held for at least 1 year in a general questionnaire and in 14 questionnaires specific to jobs and industries likely to entail exposure to suspected lymphoma carcinogens (dry cleaners, farmers or gardeners, textile workers, meat workers or slaughterers, chemical industry workers, painters, hairdressers, wood workers, printers, leather or tannery workers, teachers or others working with children, metal degreasers, health professionals and grain millers or bakers).
Wong 2012 [35]	Case-control	NHL 0.75 (0.55-1.03) B-NHL 0.73 (0.53-1.02) T-NHL 1.10 (0.55-2.21)	Age, sex, study center , month of diagnosis, race, education, housing type, BMI, history of any cancer in the first degree relatives	Participants were defined as outdoor workers if they had spent at least 30 min working outside under sun (between 9 am and 5 pm) in any of the jobs that lasted 1 year or more. Categorized participants into ‘‘indoor work only’’ workers, and those who spent all or part of their working hours outdoors ‘‘mixed indoor ± outdoor’’ workers.
Hakansson 2001 [36]	Cohort	NHL Men 1.3 (0.9-1.9)	Age, smoking, and magnetic field exposure	The occupational exposure to sunlight from outdoor work was assessed by an experienced industrial hygienist from the construction industry (N. Hallin). The hygienist classified the sunlight exposure for the job tasks into four categories with exposure scores 0, 1, 2, and 3

### Association between occupational sun exposure and risk of NHL

**Figure 2:**
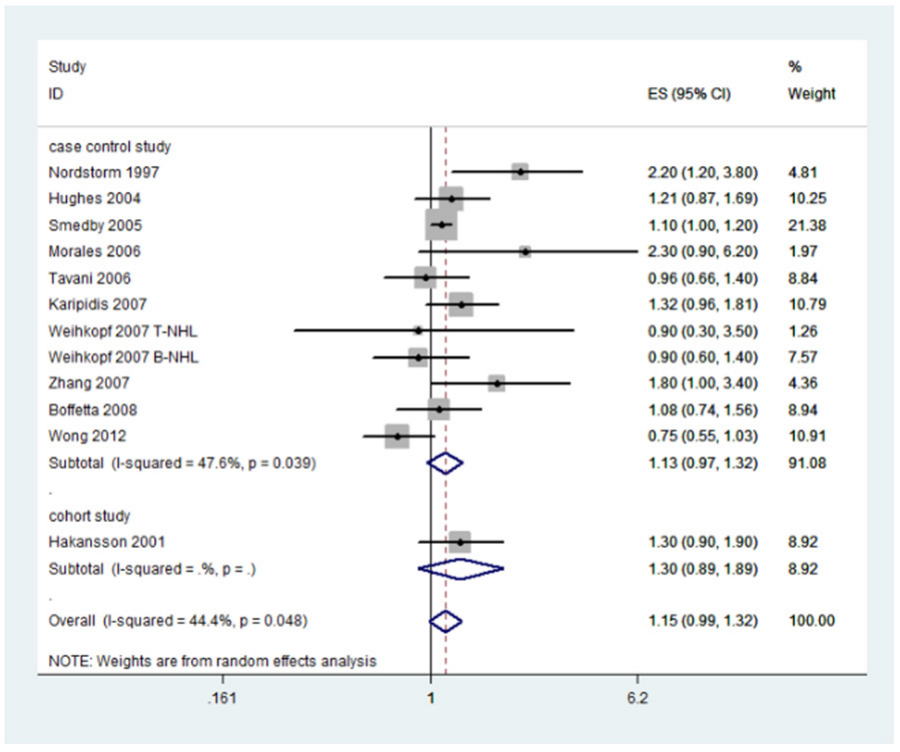
Forest plot and summary OR of the association between occupational sun exposure and risk of NHL

In the meta-analysis, the summary estimated for occupational sun exposure showed no statistical association between occupational sun exposure and risk of NHL 1.15(95%CI: 0.99, 1.32; *I^2^*=44.4%). (Figure 2) Among the 11 enrolled studies, three studies showed positive relationship between occupational sun exposure and the risk of NHL [27, 29, 33]. The ORs differ from 0.75(95%CI: 0.55, 1.03) to 2.30(95%CI: 0.96, 6.20). There was a moderate heterogeneity among these studies (*I^2^*=44.4%, p for heterogeneity =0.048), therefore we used the random-effect model to calculate the summary OR.

**Figure 3:**
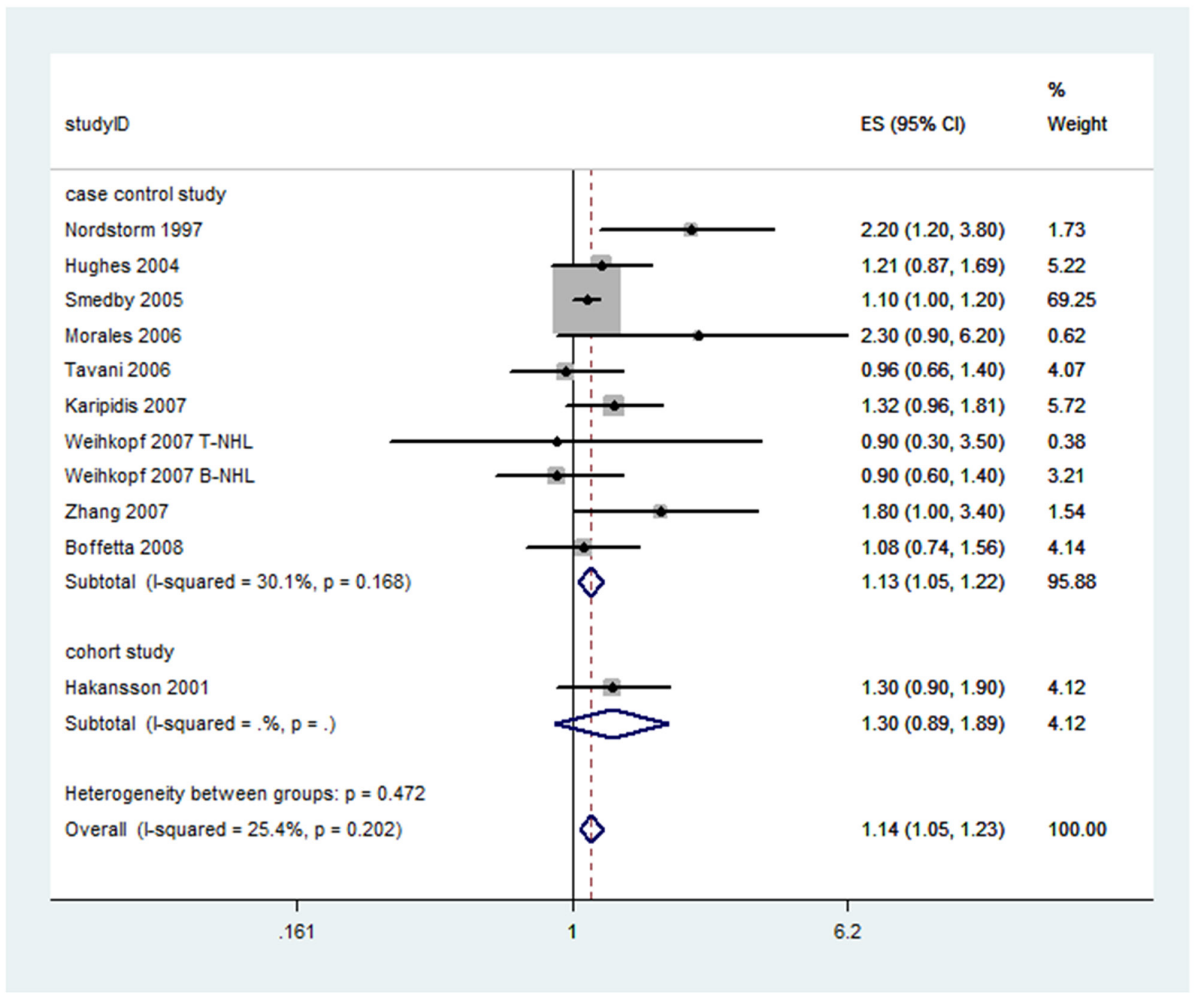
Forest plot and summary OR for Caucasian population of occupational sun exposure and risk of NHL

Ten studies reported the Caucasian population. Occupational sun exposure was positively associated with the risk of NHL 1.14 (95%CI: 1.05, 1.23; *I^2^*=25.4% p for heterogeneity =0.202). No heterogeneity was observed, thus fixed-effect model was provided. (Figure 3)

**Figure 4:**
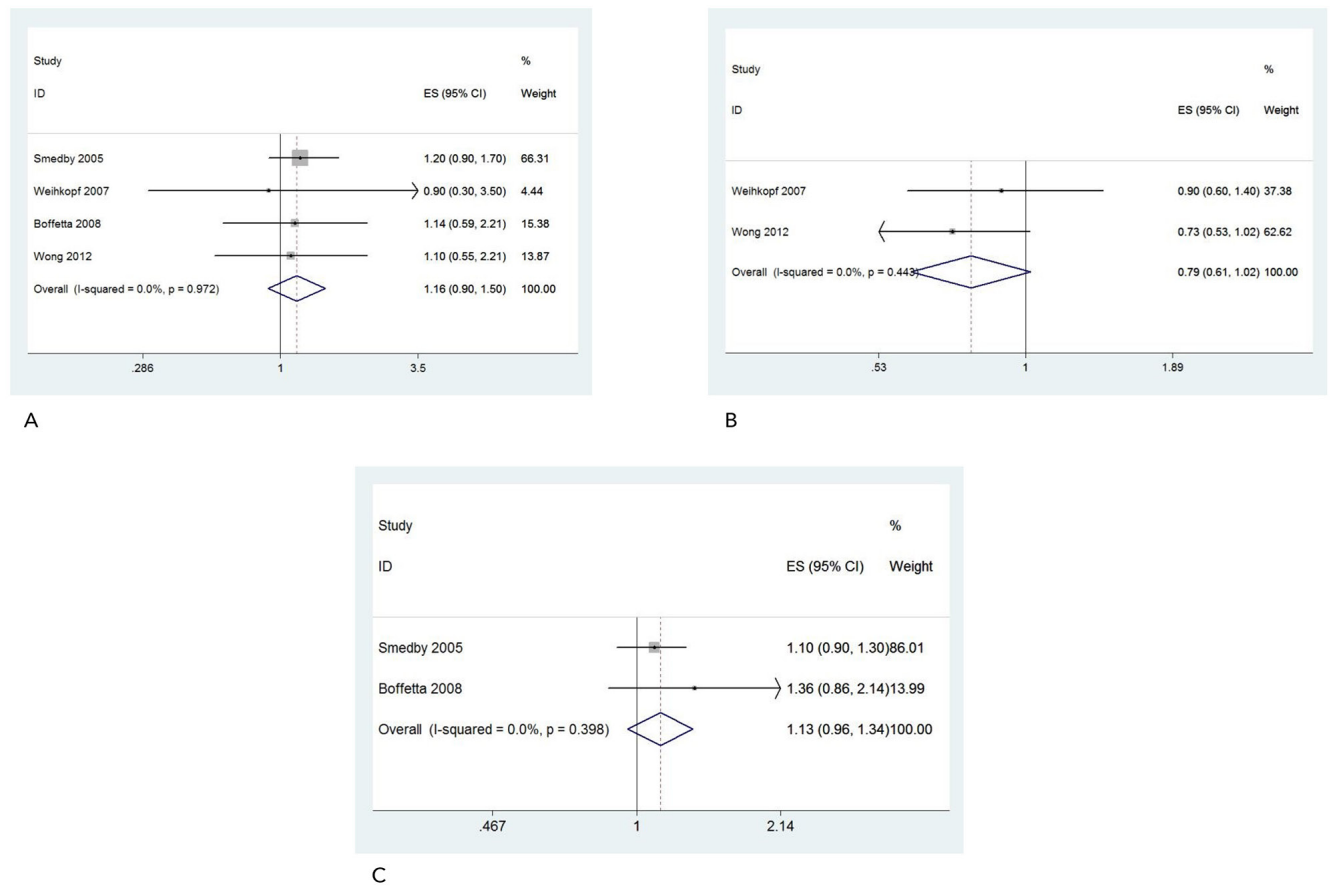
Forest plot and summary OR of T-cell NHL **(A)**, B-cell NHL **(B)** and CLL **(C)**.

Our results for occupational sun exposure did not display an association in risk for common NHL subtypes. The results of T-cell NHL (ORs: 1.16; 95%CI: 0.90, 1.50) and B-cell NHL (ORs: 0.79; 95%CI: 0.61, 1.02) analyses were presented in Figure 4A and Figure 4B. There are no association in Chronic Lymphocytic Leukemia (CLL) either. The summary ORs for CLL was 1.13 (0.96, 1.34) (Figure 4C).

**Figure 5:**
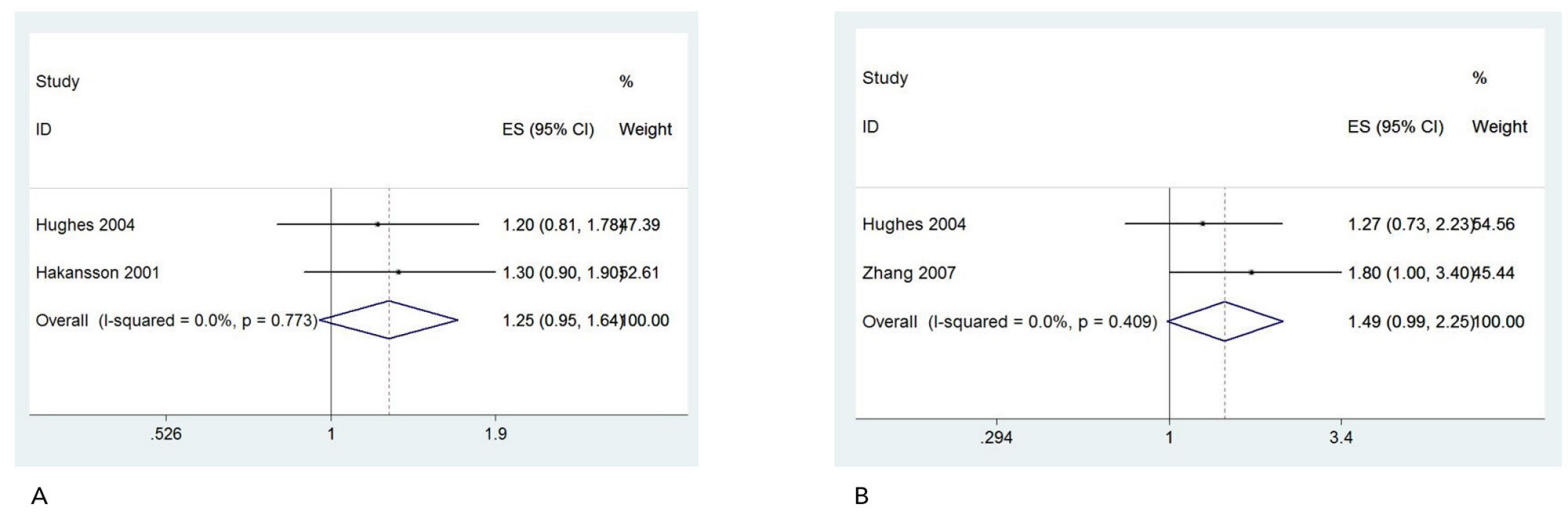
Forest plot and summary OR of male **(A)** and female **(B)**.

Gender specific information on occupational sun exposure and risk of NHL was available in three studies [28, 33, 36]. The pooled ORs were 1.25 (95%CI: 0.95, 1.64) (Figure 5A) for males and 1.49 (95%CI: 0.99, 2.25) (Figure 5B) for females. We observed no heterogeneity, so fixed-effect model was provided.

### Publication bias and sensitivity analyses

**Figure 6:**
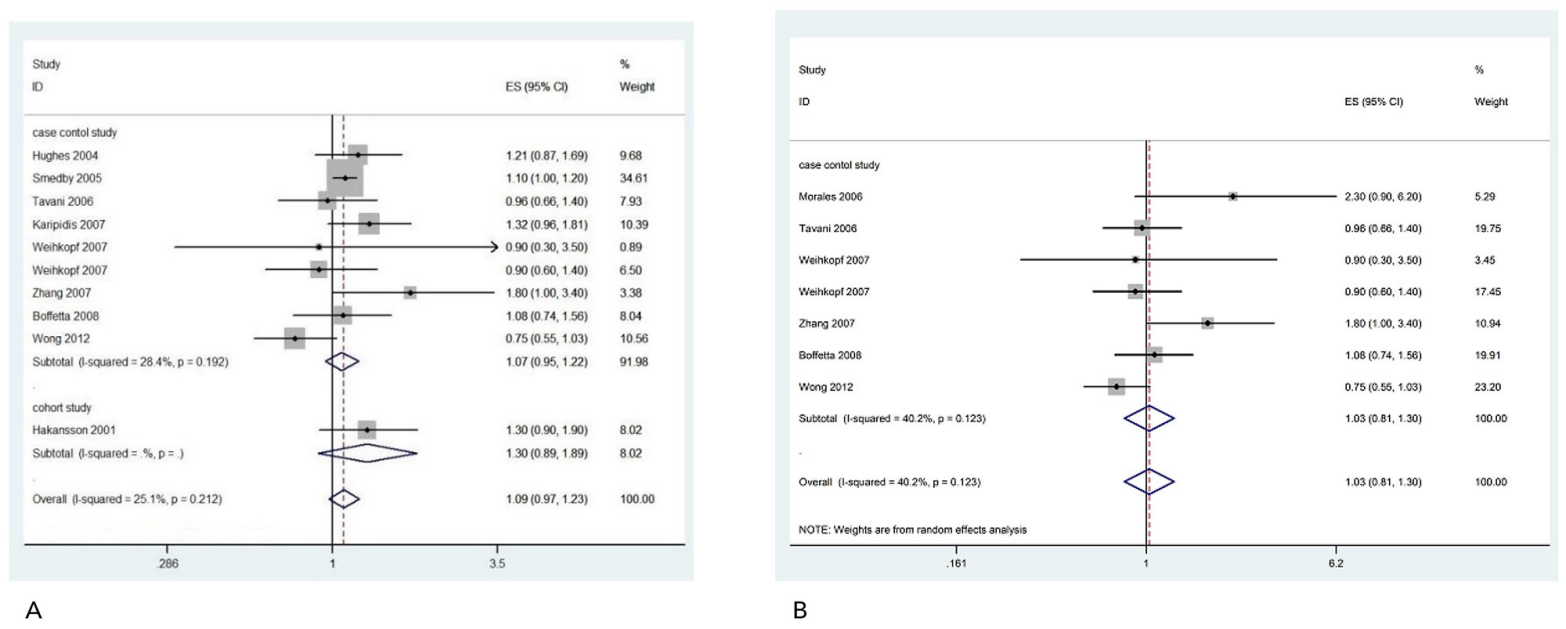
Sensitivity analysis of forest plot and summary OR of nine studies **(A)** and face to face interview studies **(B)**.

In order to evaluate the impact of potential publication bias, we applied the Begg’s test (p=0.37) and Egger’s test (p=0.37) for the association between occupational sun exposure and the risk of NHL. The results indicated no publication bias among these studies. In addition, no publication bias was detected for the positive association between occupational sun exposure and the risk of NHL in Caucasian population, either (Begg’s test: p=0.53; Egger’s test: p=0.14). To investigate heterogeneity in our meta-analysis, we evaluated sensitivity analysis within the studies. Eleven studies which included in our meta-analysis were the relationship between occupational sun exposure and risk of NHL, two studies were focused on specific types of NHL [27, 37], so we conducted a sensitivity analysis restricted to those nine studies. Results did not change when the aforementioned studies were included or excluded. The pooled OR was 1.09 (95%CI: 0.97, 1.23) with a significant decreased heterogeneity among these nine studies (*I^2^*=25.1%, p for heterogeneity =0.21 (Figure 6A). The result was consistently to the overall pooled OR, which suggested our study was reliable.

Data collection method from six studies were in person interview [30, 32-35, 37], four studies were though self-administered questionnaire and followed by a telephone interview [27-29, 31], and one study was used the data files from nationwide occupational health service organization [36]. We conducted a sensitivity analysis restricted to six in person interview studies [30, 32-35, 37].Results of pooled OR was similar 1.03(95%CI: 0.81, 1.30) (Figure 6B). There were almost no heterogeneity among studies (*I^2^*=40.2%, p for heterogeneity =0.12).

**Figure 7:**
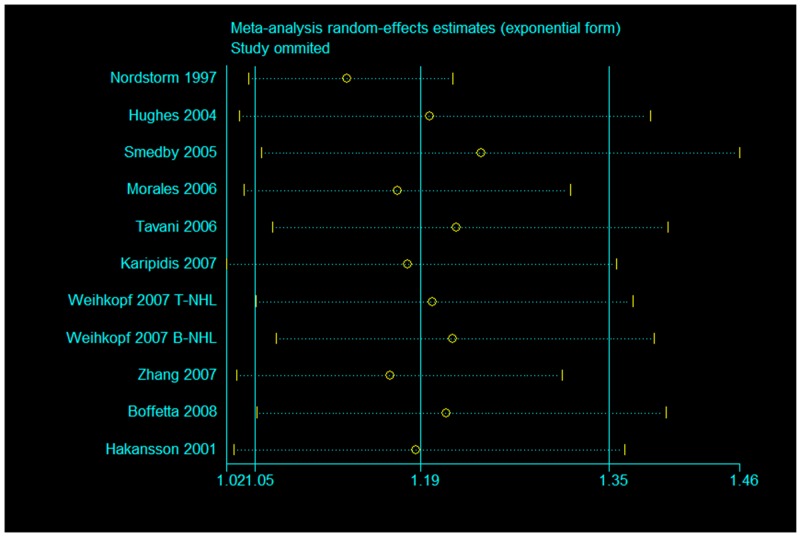
Sensitivity analysis for Caucasian population of occupational sun exposure and risk of NHL

We applied sensitivity analysis on our positive results among Caucasian population. The pooled estimate did not vary substantially with the exclusion of any single study (Figure 7).

## DISCUSSION

Our meta-analysis of 11 included case-control and cohort studies indicated that occupational ultraviolet exposure was a risk factor for NHL in Caucasian population. However, there is no association between occupational sun exposure and risk of NHL in general population as well as common NHL subtypes. In addition, gender specific study did not show any association either. Most studies included in our meta-analysis showed no association between occupational sun exposure and risk of NHL except for Nordstorm et al (OR: 2.2, 95%CI: 1.2-3.8) [27] and Zhang et al (OR: 1.8, 95%CI: 1.0-3.4) [33]. Those two studies reported occupational ultraviolet exposure increased the risk of NHL. InterLymph organization systematically analyzed ten studies about occupational UV exposure and NHL incidence in 2008, results were consistent with our main meta-analysis [38].

Our main findings showed no association between occupational ultraviolet exposure and NHL risk. When we omitted the study of Asian population our results are positively correlated. Hughes et al reported the pigmentary characteristics and NHL risk. In this study, it categorized the six ethnicity groups including Asian. They have found that the very fair skin compared to brown or olive skin had 44% increased risk of NHL [25]. Interestingly, the Singapore study was not only the Asian population study but also the only low latitudes study [35]. Several studies have yielded the same results. Reduced risk or no effect were found in mid latitudes or low latitudes [28, 38], while increased risk was found at higher latitudes [26, 39-41]. Grant WB proposes that UVA is a risk factor through impairing the immune system, while UVB is a protection factor through vitamin D production [42]. The ratio of UVA/UVB increases while latitude increases. Thus, the pooled result of our higher latitude Caucasian population studies showed that occupational UV exposure was a risk factor for NHL.

Many of the studies which included in this meta-analysis reported that there is no relationship between occupational UV exposure and NHL risk, however at the same time, reported the daily, casual UV exposure is a protective factor for NHL [28, 29, 32, 34, 35]. This result was consistent with InterLymph Organization2008 analysis [43]. One possible explanation is that most studies would have attributed any time duration occupational UV exposure cases into exposure group, while for the individual, it may be exposed to a period of time, rather than continuous exposure [28, 35, 43]. In addition, farmers often categorized into occupational UV exposure group, but some of these people at the same time contacting potential risk factors NHL such as pesticides, alkylation, etc [30, 36]. For example, Smedy et al published that Ever having an outdoor occupation for 1 year or more was associated with a slightly increased risk of non-Hodgkin lymphoma (OR = 1.2, 95% CI = 1.0 to 1.3), but this association was weakened (OR = 1.1, 95% CI = 1.0 to 1.2,) after additional adjustment for occupational exposure to pesticides [29].

As with any meta-analysis of observational studies, our study has limitations.

First, moderate heterogeneity was found across our main analysis, which can be explained by the multiple differences between studies with regard to the study designs, sample sizes, analysis strategies, participants’ baseline characteristics, adjustments for confounders and occupational history assessment methods. For example, the control source of eight studies were population based [27-29, 31-33, 36, 37], and two studies were hospital based [30, 35], while one study included both population control and hospital based control source [34]. Sample sizes were different from eleven studies included in this meta-analysis. Two studies had relatively small numbers of participants and specific NHL types [27, 37], which raised some concerns regarding the reliability of their results. Thus, we used the random-effects model to determine the overall estimate of variability.

Secondly, half of the studies in this meta-analysis relied on self-administered questionnaire, while anther half studies gathered information from interview. The participants may have different attitudes and different understanding towards questions under different methods. Besides, the total occupational sun exposure hours are varies across studies and participants. Finally, in a meta-analysis of published studies, the potential publication bias might influence the results, because studies with null results tend not to be published. Nevertheless, our publication bias test showed no possible bias.

In summary, our meta-analysis suggested that occupational UV exposure was a risk factor for NHL in Caucasian population. While, there had no relationship between occupational ultraviolet exposure and risk of NHL as well as NHL common subtypes. Besides, gender specific occupational sun exposure also indicated no association on risk of NHL.

## MATERIALS AND METHODS

### Literature search

We reported this article in accordance with MOOSE (meta-analysis of observational studies in epidemiology) guidelines [44]. We systematically searched four databases: PubMed, ISI web of science, the Cochrane Library and EMBASE for studies published in any languages (up to 2016, August 17th) using the following search items: ultraviolet radiation, ultraviolet ray, ultraviolet light, sunlight exposure or solar ultraviolet exposure combined with non-Hodgkin lymphoma or lymphoid malignancies. The search was restricted to studies of human participants. We also have reviewed the reference lists of all pertinent articles to search for more studies.

### Inclusion criteria

To be included in this meta-analysis, studies had to have met the following criteria: (1) NHL cases were medically confirmed by histopathology diagnoses; (2) the study was designed as case-control or cohort study; (3) the occupational sunlight exposure and incidence of NHL were associated; (4) detailed data of odds ratios (ORs) or relative risks (RRs) with 95% confidence interval (CI); (5) All the cases were adult (age≥17 years old). We did not include the studies that only report the death rate of NHL without incidence rate. When there were multiple published reports from the same study population, the most recent or the most informative report was selected for analysis.

### Data extraction

We extracted the following information from each study: authors’ name, year of publication, study type, study location, sample size (numbers of case patients and control subjects), study period, participation’s age, type of control source, assessment of data collection, and statistical covariates adjustment in the analysis. ORs or RRs with corresponding 95%CIs for each study were either extracted directly from the article or calculated from available raw data.

### Statistical analysis

To pool the results of individual studies together, we used a general variance-based method in the meta-analysis. The multivariate adjusted ORs and 95% CIs presented in the literature were used. In situations where the incidence is low, the odds ratio approximates the relative risk, therefore, in looking at studies of NHL (a rare condition), it is acceptable to compare OR and RR estimates [45]. The outcomes are presented as a forest plot with 95% CIs.

Statistical heterogeneity among studies was tested with the Q statistic, and statistical inconsistency was quantified with the *I^2^* statistic [46]. When *I^2^* was from 0% to 40% along with *p* >0.10 the heterogeneity might not be important. If the meta-analysis has no heterogeneity, fixed-effects model with the Mantel-Haeszel method would be used to combine the individual studies [47], otherwise, the random-effects method (DerSimonian 1986) was used for pooling [48].

There were two studies focused only on specific types of NHL [27, 37]. We further conducted a sensitivity analysis restricted to the rest nine studies to evaluate the stability of the pooled estimates between occupational sun exposure and risk of NHL.

The Egger’s test and Begg’s test were used to assess for publication bias [49, 50]. P<0.05 was considered statistically significant publication bias. All statistical analyses was performed by using STATA (version 11.0; StataCorp, College Station, TX).

## References

[1] Zelenetz AD, Abramson JS, Advani RH, Andreadis CB, Byrd JC, Czuczman MS, Fayad L, Forero A, Glenn MJ, Gockerman JP, Gordon LI, Harris NL, Hoppe RT (2010). NCCN Clinical Practice Guidelines in Oncology: non-Hodgkin’s lymphomas. J Natl Compr Canc Netw.

[2] Jemal A, Siegel R, Xu J, Ward E (2010). Cancer statistics, 2010. CA Cancer J Clin.

[3] UK cancer incidence. Cancer Research UK (2010). http://www.cancerresearchuk.org/cancer-info/cancerstats/incidence/.

[4] Zheng T, Mayne ST, Boyle P, Holford TR, Liu WL, Flannery J (1992). Epidemiology of non-Hodgkin lymphoma in Connecticut. 1935-1988. Cancer.

[5] Espinosa A, Zock JP, Benavente Y, Boffetta P, Becker N, Brennan P, Cocco P, Foretova L, Maynadié M, Staines A, Nieters A, Kogevinas M, de Sanjose S (2013). Occupational exposure to immunologically active agents and risk for lymphoma: the European Epilymph case-control study. Cancer Epidemiol.

[6] Iscovich J, Paltiel O, Azizi E, Kuten A, Gat A, Lifzchitz-Mercer B, Zlotogorski A, Polliack A (1998). Cutaneous lymphoma in Israel, 1985-1993: a population-based incidence study. Br J Cancer.

[7] Karunanayake CP, McDuffie HH, Dosman JA, Spinelli JJ, Pahwa P (2008). Occupational exposures and non-Hodgkin’s lymphoma: canadian case-control study. Environ Health.

[8] Karunanayake CP, Singh GV, Spinelli JJ, McLaughlin JR, Dosman JA, McDuffie HH, Pahwa P (2009). Occupational exposures and Hodgkin Lymphoma: canadian case-control study. J Occup Environ Med.

[9] McDuffie HH, Pahwa P, Robson D, Dosman JA, Fincham S, Spinelli JJ, McLaughlin JR (2005). Insect repellents, phenoxyherbicide exposure, and non-Hodgkin’s lymphoma. J Occup Environ Med.

[10] Purdue MP, Freedman DM, Gapstur SM, Helzlsouer KJ, Laden F, Lim U, Maskarinec G, Rothman N, Shu XO, Stevens VL, Zeleniuch-Jacquotte A, Albanes D, Bertrand K (2010). Circulating 25-hydroxyvitamin D and risk of non-hodgkin lymphoma: Cohort Consortium Vitamin D Pooling Project of Rarer Cancers. Am J Epidemiol.

[11] Kelly JL, Friedberg JW, Calvi LM, van Wijngaarden E, Fisher SG (2010). A case-control study of ultraviolet radiation exposure, vitamin D, and lymphoma risk in adults. Cancer Causes Control.

[12] Hartge P, Lim U, Freedman DM, Colt JS, Cerhan JR, Cozen W, Severson RK, Davis S (2006). Ultraviolet radiation, dietary vitamin D, and risk of non-Hodgkin lymphoma (United States). Cancer Causes Control.

[13] Soni LK, Hou L, Gapstur SM, Evens AM, Weisenburger DD, Chiu BC (2007). Sun exposure and non-Hodgkin lymphoma: a population-based, case-control study. Eur J Cancer.

[14] Grandin L, Orsi L, Troussard X, Monnereau A, Berthou C, Fenaux P, Marit G, Soubeyran P, Huguet F, Milpied N, Leporrier M, Hemon D, Clavel J (2008). UV radiation exposure, skin type and lymphoid malignancies: results of a French case-control study. Cancer Causes Control.

[15] Kane EV, Painter D, Roman E, Allan J, Law G, Lightfoot T (2010). Melanocortin 1 receptor (MC1R), pigmentary characteristics and sun exposure: findings from a case-control study of diffuse large B-cell and follicular lymphoma. Cancer Epidemiol.

[16] Kelly JL, Drake MT, Fredericksen ZS, Asmann YW, Liebow M, Shanafelt TD, Feldman AL, Ansell SM, Macon WR, Herr MM, Wang AH, Nowakowski GS, Call TG (2012). Early life sun exposure, vitamin D-related gene variants, and risk of non-Hodgkin lymphoma. Cancer Causes Control.

[17] Bertrand KA, Chang ET, Abel GA, Zhang SM, Spiegelman D, Qureshi AA, Laden F (2011). Sunlight exposure, vitamin D, and risk of non-Hodgkin lymphoma in the Nurses’ Health Study. Cancer Causes Control.

[18] Chang ET, Canchola AJ, Cockburn M, Lu Y, Wang SS, Bernstein L, Clarke CA, Horn-Ross PL (2011). Adulthood residential ultraviolet radiation, sun sensitivity, dietary vitamin D, and risk of lymphoid malignancies in the California Teachers Study. Blood.

[19] Lin SW, Wheeler DC, Park Y, Cahoon EK, Hollenbeck AR, Freedman DM, Abnet CC (2012). Prospective study of ultraviolet radiation exposure and risk of cancer in the United States. Int J Cancer.

[20] Freedman DM, Zahm SH, Dosemeci M (1997). Residential and occupational exposure to sunlight and mortality from non-Hodgkin’s lymphoma: composite (threefold) case-control study. BMJ.

[21] Grant WB (2002). An estimate of premature cancer mortality in the U.S. due to inadequate doses of solar ultraviolet-B radiation. Cancer.

[22] Langford IH, Bentham G, McDonald AL (1998). Mortality from non-Hodgkin lymphoma and UV exposure in the European Community. Health Place.

[23] Simard JF, Baecklund F, Chang ET, Baecklund E, Hjalgrim H, -Olov Adami H, Glimelius B, Smedby KE (2013). Lifestyle factors, autoimmune disease and family history in prognosis of non-hodgkin lymphoma overall and subtypes. Int J Cancer.

[24] van Wijngaarden E, Savitz DA (2001). Occupational sunlight exposure and mortality from non-Hodgkin lymphoma among electric utility workers. J Occup Environ Med.

[25] Hughes AM, Armstrong BK, Vajdic CM, Turner J, Grulich A, Fritschi L, Milliken S, Kaldor J, Benke G, Kricker A (2004). Pigmentary characteristics, sun sensitivity and non-Hodgkin lymphoma. Int J Cancer.

[26] Adami J, Gridley G, Nyrén O, Dosemeci M, Linet M, Glimelius B, Ekbom A, Zahm SH (1999). Sunlight and non-Hodgkin’s lymphoma: a population-based cohort study in Sweden. Int J Cancer.

[27] Nordström M, Hardell L, Magnusson A, Hagberg H, Rask-Andersen A (1997). Occupation and occupational exposure to UV light as risk factors for hairy cell leukaemia evaluated in a case-control study. Eur J Cancer Prev.

[28] Hughes AM, Armstrong BK, Vajdic CM, Turner J, Grulich AE, Fritschi L, Milliken S, Kaldor J, Benke G, Kricker A (2004). Sun exposure may protect against non-Hodgkin lymphoma: a case-control study. Int J Cancer.

[29] Smedby KE, Hjalgrim H, Melbye M, Torrång A, Rostgaard K, Munksgaard L, Adami J, Hansen M, Porwit-MacDonald A, Jensen BA, Roos G, Pedersen BB, Sundström C (2005). Ultraviolet radiation exposure and risk of malignant lymphomas. J Natl Cancer Inst.

[30] Tavani A, Bosetti C, Franceschi S, Talamini R, Negri E, La Vecchia C (2006). Occupational exposure to ultraviolet radiation and risk of non-Hodgkin lymphoma. Eur J Cancer Prev.

[31] Karipidis KK, Benke G, Sim MR, Kauppinen T, Kricker A, Hughes AM, Grulich AE, Vajdic CM, Kaldor J, Armstrong B, Fritschi L (2007). Occupational exposure to ionizing and non-ionizing radiation and risk of non-Hodgkin lymphoma. Int Arch Occup Environ Health.

[32] Weihkopf T, Becker N, Nieters A, Mester B, Deeg E, Elsner G, Blettner M, Seidler A (2007). Sun exposure and malignant lymphoma: a population-based case-control study in Germany. Int J Cancer.

[33] Zhang Y, Holford TR, Leaderer B, Boyle P, Zhu Y, Wang R, Zou K, Zhang B, Wise JP, Qin Q, Kilfoy B, Han J, Zheng T (2007). Ultraviolet radiation exposure and risk of non-Hodgkin’s lymphoma. Am J Epidemiol.

[34] Boffetta P, van der Hel O, Kricker A, Nieters A, de Sanjosé S, Maynadié M, Cocco PL, Staines A, Becker N, Font R, Mannetje A, Goumas C, Brennan P (2008). Exposure to ultraviolet radiation and risk of malignant lymphoma and multiple myeloma--a multicentre European case-control study. Int J Epidemiol.

[35] Wong KY, Tai BC, Chia SE, Kuperan P, Lee KM, Lim ST, Loong S, Mow B, Ng SB, Tan L, Tan SY, Tan SH, Tao M (2012). Sun exposure and risk of lymphoid neoplasms in Singapore. Cancer Causes Control.

[36] Håkansson N, Floderus B, Gustavsson P, Feychting M, Hallin N (2001). Occupational sunlight exposure and cancer incidence among Swedish construction workers. Epidemiology.

[37] Morales-Suárez-Varela MM, Olsen J, Johansen P, Kaerlev L, Guénel P, Arveux P, Wingren G, Hardell L, Ahrens W, Stang A, Llopis A, Merletti F, Guillen-Grima F, Masala G (2006). Occupational sun exposure and mycosis fungoides: a European multicenter case-control study. J Occup Environ Med.

[38] Petridou ET, Dikalioti SK, Skalkidou A, Andrie E, Dessypris N, Trichopoulos D, Childhood Hematology-Oncology Group (2007). Sun exposure, birth weight, and childhood lymphomas: a case control study in Greece. Cancer Causes Control.

[39] Grant WB (2012). Role of solar UVB irradiance and smoking in cancer as inferred from cancer incidence rates by occupation in Nordic countries. Dermatoendocrinol.

[40] Bentham G (1996). Association between incidence of non-Hodgkin’s lymphoma and solar ultraviolet radiation in England and Wales. BMJ.

[41] Newton R, Roman E, Fear N, Carpenter L (1996). Non-Hodgkin’s lymphoma and solar ultraviolet radiation. Data are inconsistent. BMJ.

[42] Grant WB (2012). Ultraviolet exposure and non-Hodgkin’s lymphoma: beneficial and adverse effects?. Cancer Causes Control.

[43] Kricker A, Armstrong BK, Hughes AM, Goumas C, Smedby KE, Zheng T, Spinelli JJ, De Sanjosé S, Hartge P, Melbye M, Willett EV, Becker N, Chiu BC, Interlymph Consortium (2008). Personal sun exposure and risk of non Hodgkin lymphoma: a pooled analysis from the Interlymph Consortium. Int J Cancer.

[44] Stroup DF, Berlin JA, Morton SC, Olkin I, Williamson GD, Rennie D, Moher D, Becker BJ, Sipe TA, Thacker SB (2000). Meta-analysis of observational studies in epidemiology: a proposal for reporting. Meta-analysis Of Observational Studies in Epidemiology (MOOSE) group. JAMA.

[45] Greenland S (1987). Quantitative methods in the review of epidemiologic literature. Epidemiol Rev.

[46] Higgins JP, Thompson SG (2002). Quantifying heterogeneity in a meta-analysis. Stat Med.

[47] Mantel N, Haenszel W (1959). Statistical aspects of the analysis of data from retrospective studies of disease. J Natl Cancer Inst.

[48] DerSimonian R, Laird N (1986). Meta-analysis in clinical trials. Control Clin Trials.

[49] Egger M, Davey Smith G, Schneider M, Minder C (1997). Bias in meta-analysis detected by a simple, graphical test. BMJ.

[50] Begg CB, Mazumdar M (1994). Operating characteristics of a rank correlation test for publication bias. Biometrics.

